# Extracellular Matrix Protein Lumican Promotes Clearance and Resolution of *Pseudomonas aeruginosa* Keratitis in a Mouse Model

**DOI:** 10.1371/journal.pone.0054765

**Published:** 2013-01-24

**Authors:** Hanjuan Shao, Sherri-Gae Scott, Chiaki Nakata, Abdel R. Hamad, Shukti Chakravarti

**Affiliations:** 1 Department of Medicine, Johns Hopkins School of Medicine, Baltimore, Maryland, United States of America; 2 Department of Pathology, Johns Hopkins School of Medicine, Baltimore, Maryland, United States of America; 3 Department of Cell Biology, Johns Hopkins School of Medicine, Baltimore, Maryland, United States of America; 4 Department of Ophthalmology, Johns Hopkins School of Medicine, Baltimore, Maryland, United States of America; Cedars-Sinai Medical Center, United States of America

## Abstract

Lumican is an extracellular protein that associates with CD14 on the surface of macrophages and neutrophils, and promotes CD14-TLR4 mediated response to bacterial lipopolysaccharides (LPS). Lumican-deficient (*Lum* ^−/−^) mice and macrophages are impaired in TLR4 signals; raising the possibility that lumican may regulate host response to live bacterial infections. In a recent study we showed that *in* *vitro Lum* ^−/−^ macrophages are impaired in phagocytosis of gram-negative bacteria and in a lung infection model the *Lum* ^−/−^ mice showed poor survival. The cornea is an immune privileged barrier tissue that relies primarily on innate immunity to protect against ocular infections. Lumican is a major component of the cornea, yet its role in counteracting live bacteria in the cornea remains poorly understood. Here we investigated Pseudomonas aeruginosa infections of the cornea in *Lum* ^−/−^ mice. By flow cytometry we found that 24 hours after infection macrophage and neutrophil counts were lower in the cornea of *Lum* ^−/−^ mice compared to wild types. Infected *Lum* ^−/−^ corneas showed lower levels of the leukocyte chemoattractant CXCL1 by 24–48 hours of infection, and increased bacterial counts up to 5 days after infection, compared to *Lum^+/−^* mice. The pro-inflammatory cytokine TNF-α was comparably low 24 hours after infection, but significantly higher in the *Lum* ^−/−^ compared to *Lum* ^+/−^ infected corneas by 2–5 days after infection. Taken together, the results indicate that lumican facilitates development of an innate immune response at the earlier stages of infection and lumican deficiency leads to poor bacterial clearance and resolution of corneal inflammation at a later stage.

## Introduction

Lumican is an extracellular matrix proteoglycan that is known to regulate collagen fibril structure and corneal transparency [1], [2]. Our recent work and those of others suggest that lumican regulates wound healing and innate immune responses [3]–[7]. The lumican core protein carries tandem repeats of leucine rich motifs, a feature shared by pathogen recognition receptors [8], that prompted us to investigate recognition of pathogen associated molecular patterns (PAMPs) by lumican [3]. We found that lumican interacts with bacterial lipopolysaccharide (LPS) endotoxins and facilitates host response to LPS. Mice deficient in lumican (*Lum* ^−/−^) were hypo responsive to LPS and compared to wild types showed improved survival in a septic shock model induced by intraperitoneal injection of LPS [3]. LPS sensing is mediated by toll-like receptor (TLR) 4; the sequence of events upon LPS encounter involves its binding to LBP (LPS-binding protein), subsequent binding to CD14, a glycosylphosphatidyl inositol linked adaptor protein, which delivers LPS to MD-2, a soluble protein, complexed to TLR4, causing a conformational change in TLR4, recruitment of cytoplasmic adaptor proteins and signal transduction [8]. We identified binding between lumican and CD14, and determined that recombinant lumican could rescue LPS signaling in *Lum* ^−/−^ peritoneal macrophages [3], [5]. Peritoneal macrophages from lumican knockout mice were also impaired in *in vitro* phagocytosis of gram-negative bacteria in a CD14-dependent mechanism. These LPS signaling deficiencies raise the possibility that challenged with live bacteria, the lumican-null mice may manifest specific host defense susceptibilities.

We have minimally examined lumican functions in a live bacterial infection setting in a lung infection model where the *Lum* ^−/−^ mice showed increased bacterial persistence and poor host survival [5]. In the cornea lumican may have a particularly important and unique role in bacterial defense for two reasons. First, lumican, together with other small leucine-rich repeat proteoglycans (SLRPs), decorin, biglycan, keratocan and collagen types I, III and V, is a major component of the cornea [9]. Second, the cornea relies heavily on innate immune functions for barrier protection as adaptive immunity is restricted to ensure its immune privileged status, limit inflammation and maintain clarity [10], [11]. TLR4 and 5 are active regulators of innate immunity in the cornea, and shown to promote infiltration of polymorphonuclear leukocytes or neutrophils (PMN) and macrophages into the stroma, to up regulate cytokines and chemokines, but unabated TLR signals can cause inflammation-associated opacity [12], [13]. While lumican has been investigated in the context of sterile LPS keratitis [14], [15], its role in host response to live bacterial challenge remains poorly understood.

In the current study we investigated the role of lumican in bacterial keratitis. We selected gram-negative *Pseudomonas aeruginosa* as the bacterium of choice as it is frequently an opportunistic pathogen that causes an ulcerative and vision debilitating keratitis in humans [16]. Predisposing risk factors for bacterial keratitis vary with geographic location; in developing countries it is primarily linked to non-surgical traumas [17], whereas, in the United States it is associated with extended-wear contact lenses [18], [19], photorefractive keratectomy and keratoplasty [20]–[22]. Disruptions in the mucus barrier of the cornea allow *P.aeruginosa* to enter and traverse the corneal epithelium, and reach the stroma. Bacterial encounter elicits an innate immune response from the host that involves toll-like receptor (TLR) 4, 5 and 9 to bacterial lipopolysaccharide endotoxins, flagellin and bacterial DNA, respectively [8], [13], [23]–[25]. In *P.aeruginosa* infections of the cornea, tissue damage ensues initially from bacterial activities [26]–[28], and subsequently from host inflammatory response, inflammatory cell infiltrates, release of proteinases and extracellular matrix (ECM) degradation [16], [29]. While many components of the host innate and adaptive immune responses are being investigated in *P.aeruginosa*-keratitis [10], the role of stromal ECM components in this regard is just beginning to be elucidated. Our findings show increased survival of bacteria and un-resolved inflammation with associated bystander corneal tissue damage in infected *Lum* ^−/−^ corneas.

## Materials and Methods

### *P. aeruginosa* Strains and Growth Conditions

The *P. aeruginosa* strain 19660 (ATCC, Manassas, VA) was used for the murine keratitis model. *P. aeruginosa* strain 808 (gift from Dr. David P. Speert, University of British Columbia and BC Children’s Hospital, Canada), which is taken up by the host in a CD14 dependent manner, was used in the *in* *vitro* killing assays. The *P. aeruginosa* strains were incubated overnight in Cetrimide agar (Fluka Analytical, Sigma-Aldrich) at 37°C. Colonies were washed with 1xPBS and centrifuged at 5000 rpm for 10 minutes, and then resuspended in 1xPBS. Colony forming units (CFU) were quantified by plating dilutions onto Cetrimide agar.

### Infection of the Cornea

*Lum* ^+/−^ and *Lum* ^−/−^ mice on a C57BL/6J background, 8–10 weeks of age, were generated as we described before [1]. All animals were housed in a specific pathogen-free mouse facility at Johns Hopkins University, according to protocols approved by the Animal Care and Use Committee. Animals were treated in adherence to the ARVO Statement. The *P. aeruginosa* strain 19660 was used for the murine keratitis model. Anesthetized mice (350 mg/Kg bodyweight of 2-2-2 Tribromoethanol or Avertin®, Sigma-Aldrich, St. Louis, MO), were placed under the microscope, and the cornea of the right eye was wounded with three 1 µm incisions with a sterile 26-gauge needle such that the scratches had invaded the top 1/10^th^ of the stroma [30]. We have previously examined incision wounds of the wild type and *Lum* ^−/−^ corneas exposed to saline only and found no increase in inflammatory cell infiltration, and basal levels of the cytokines IL-1β, TNFα and IL-6 [31]in the saline controls. An aliquot of 2×10^4^ CFU of *P. aeruginosa* in 5 µL PBS was applied to the scarified cornea, and the mice were undisturbed for 30 min. Mice without any treatment were set as controls. The eyes were inspected microscopically and photographed using a Nikon Eclipse E400 Microscope with a Nikon Digital DXM 1200 Camera.

Corneal disease was scored daily in a blinded manner for 6 days after *P. aeruginosa* infection for each group of mice (3–4 mice for each group) using a scoring mechanism described earlier [32]. Score 0: eye identical with the uninfected contra lateral control eye; Score 1: faint opacity partially covering the pupil; Score 2: dense opacity covering the pupil; Score 3: dense opacity covering the entire anterior segment; Score 4: perforation of the cornea, phthisis bulbi, or both. Slit lamp photography was used to illustrate the disease.

### Quantification of Bacteria

Whole eyes were homogenized in 0.5 ml PBS on ice and serial dilutions were plated on Cetrimide Agar plates. After overnight incubation at 37°C, the number of individual colonies on plates was counted and presented as CFU per ml.

Alternatively, the surface of the cornea was sampled for the presence of bacteria in the following way. A sterile filter (2 mm diameter) was placed over the central cornea of anesthetized mice and then resuspended in 300 µL of sterile PBS. Serial 10-fold dilutions of the filter washes were plated to obtain CFU per ml.

### Quantitative RT-PCR

Whole corneas from uninfected and infected mice (n = 4 per time point) were harvested and homogenized in 800 µl TRIzol with 5 mm stainless steel beads in a TissueLyser LT small bead mill (Qiagen, Valencia, CA) at 4°C and total RNA was isolated using the Qiagen RNeasy Miniprep Kit. One microgram of each RNA sample was reverse transcribed to cDNA using Superscript II Reverse Transcriptase Kit (Applied Biosystems Life Technologies, Carlsbad, CA). Quantitative RT-PCR was performed with SYBR Green PCR Master Mix Taqman 7900HT System (Applied Biosystems). We designed the following primers (IDT Integrated DNA Technologies, Coralville, IA) using Primer 3 software for mouse *Cxcl1* 5′-ACTGCACCCAAACCGAAGTC-3′ forward and 5′- CAAGGGAGCTTCAGGGTCAA-3′ reverse, *Lum* 5′-TCGAGCTTGATCTCTCCTAT-3′ forward and 5′-TGGTCCCAGGTCTTACAGAA-3′ reverse, 18S 5′-GTAACCCGTTGAACCCCATT-3′ forward and 5′-CCATCCAATCGGTAGTAGCG-3′ reverse. Target genes were amplified in 25 µl reaction volume for 1 min at 95°C followed by 40 cycles of 30 seconds at 95°C, 30 seconds at 58°C and 1 min at 72°C and a final extension for 10 min at 72°C. PCR reactions were analyzed in quadruplicate, using the SDS 2.3 software (Applied Biosystems) and mRNA fold changes were calculated after normalizing to 18S RNA.

### ELISA Analysis of Cytokines

Total protein was extracted from individual corneas by homogenization using a TissueLyser LT small bead mill for 30 min in 200 µL T-PER (Thermo Scientific Pierce Protein Biology Products, Rockford, IL) and centrifuged at 13,000 rpm for 10 min. Protein concentration of the supernatant was determined by BCA assay (Thermo Fisher Scientific) and the samples were stored in aliquots at −80°C until use. TNF-α, IL-12, IL-1β and CXCL1 concentrations were measured by ELISA (R & D Systems; Minneapolis, MN) according to manufacturer’s instructions.

### Histology

Whole mouse eyes were harvested and fixed for 48 hrs in fresh 4% Methanol-free Formaldehyde Solution (Thermo Scientific) at room temperature (RT), paraffin – embedded and sectioned. The slides were stained with H&E by the Johns Hopkins Pathology Core. Images were taken in a Zeiss AXIO Observer A1 Microscope (Carl Zeiss) with an Olympus DP72 Camera (Olympus Imaging America).

### Flow Cytometry

Five whole eyes from infected and control mice were harvested and rinsed in cold PBS. Corneal cells were isolated using a modified protocol described previously (Sun et. al., 2010). Residual iris material was removed and five corneas were rinsed, pooled, minced and incubated at 37°C in 500 µl of Collagenase L/PBS (Sigma-Aldrich) for 75 mins with shaking at 125 rpm. The released cells were centrifuged, washed and resuspended in 1 ml FACS buffer (Ca^2+^/Mg ^2+^ free 1x PBS and 1% FBS), and viable cell count was established by Trypan Blue exclusion. The cells were blocked and immunostained for 30 minutes on ice using the following antibodies: PE-F4/80 (eBioscience, San Diego, CA), PerCP-Gr-1(eBioscience) and APC-Annexin V (eBioscience). Flow cytometry was performed on a FACS Calibur (Becton Dickinson &Co., San Jose, CA), using the FCS Express Version 3 software.

### *In vitro* Killing by Peritoneal Neutrophils

Mice were injected with 1 ml of 9% Casein solution (Sigma), and PMNs were harvested by peritoneal lavage 3 hours after the second injection and stored in cold 1xPBS and 0.02% EDTA as described earlier [4]. The PMNs were purified by Histopaque 1077 and 1119 (Sigma-Aldrich) density gradient cell separation as previously described [4]. The PMNs were plated in serum - free RPMI (Invitrogen) in 96 well plates (Corning) at a concentration of 2×10^5^ cells/ml in 100 µl and incubated overnight at 37°C. The concentration of *P. aeruginosa* was adjusted to 1×10^4^ CFU/3.3 µL in PBS and added to each well without prior opsonization and incubated for 30, 60 and 120 minutes. Viable CFU at the time of the experiment was determined by plating dilutions of the bacterial suspension onto Cetrimide agar plates. Bacterial killing was measured as viable CFU recovered and quantified on Cetrimide agar plates from lysed PMNs and the supernatant.

### Statistical Analysis

*P* values were ascertained using unpaired, two-tailed student’s *t*- tests using the GraphPad Prism 4 software. Data were considered as significantly different at *p*≤0.05.

## Results

### Increased Disease Score and Bacterial Load in *Lum* ^−/−^ Mice

**Figure 1:**
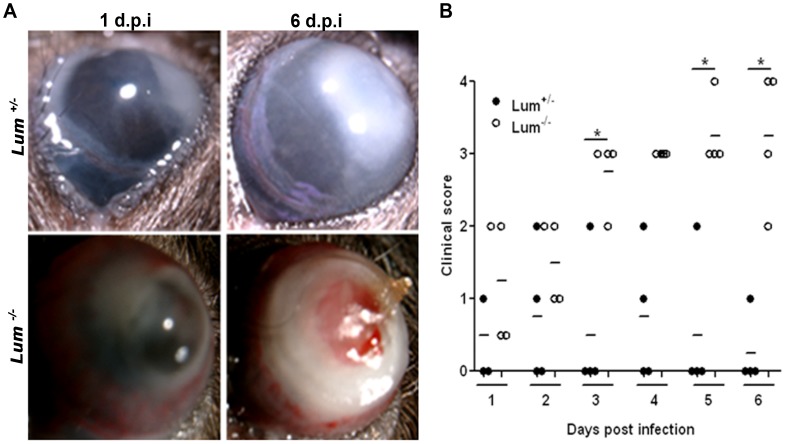
Increased clinical score in *P.aeruginosa* infected *Lum* ^−/−^ mice. Mice were infected with 2×10^4^ CFU *P.aeruginosa* ATCC19660 in one eye and scored in a blinded manner for 6 days. A representative image of infected eyes 1 and 6 days post infection (d.p.i) shown for each genotype indicates relatively similar severity on day 1 but increased opacity and damage in the *Lum* ^−/−^ cornea on 6 d.p.i (A). Daily disease scores (B) of individual animals shows increased average scores for infected *Lum* ^−/−^ corneas.* *p*≤0.05.

The course of *P. aeruginosa* keratitis in the *Lum* ^−/−^ was compared to *Lum* ^+/−^ mice, used as a lumican expressing control group. There were no major differences in the appearance of *Lum* ^+/−^ and *Lum* ^−/−^ infected eyes one day after infection; after 6 days the *Lum* ^−/−^ infected eye became suppurative and opaque in most animals (Fig. 1A). By contrast, visible signs of infection subsided by 2–5 days in the *Lum* ^+/−^ group. The mice were scored in a blinded manner every 24 hours (hrs) for six days for clinical appearance of disease (Fig. 1B). We should point out that the *Lum* ^−/−^ mice have diffused cloudy corneas resulting from increased backscattering of light due to collagen fibril disorganization, detectable by slit lamp biomicroscopy or high resolution confocal microscopy [1], [2], however, this does not interfere with scoring the mice for infection-associated corneal opacity. Clinical disease scores were generally similar in *Lum* ^−/−^ and *Lum* ^+/−^ around 24 hrs of infection. Over time however, the *Lum* ^−/−^ mice showed higher disease scores and poor resolution of corneal opacity.

**Figure 2:**
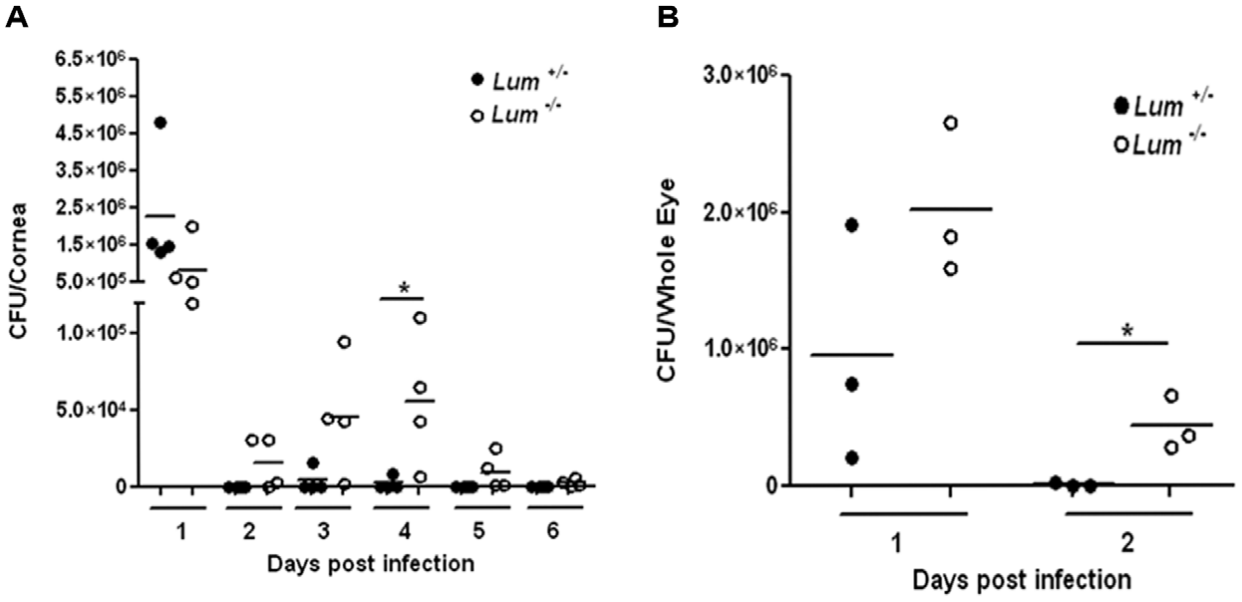
Poor clearance of *P.aeruginosa* in infected *Lum ^−/−^* corneas. Viable bacterial CFU were quantified from infected *Lum ^+/−^* and *Lum* ^−/−^ corneas. CFU were higher in ocular surfaces sampled with filter lifts (A) and whole eye homogenates (B) during the infection. Both methods yielded significantly higher CFU counts from *Lum ^−/−^* corneas as shown at day 4 (cornea surface lift) and day 2 (whole eye homogenate). *p≤0.05.

We examined bacterial load per infected eye using two different approaches. In the first, we sampled bacterial counts at the ocular surface using small circular filters, daily for 6 days (Fig. 2A). Viable bacterial yield from filters lifted off the surface 24 hrs after infection was comparable between *Lum* ^+/−^ and *Lum* ^−/−^ animals. However, by 48 hrs, three of the four *Lum* ^+/−^ mice had cleared the infection while the *Lum* ^−/−^ mice continued to yield viable bacteria for up to 6 days after infection. Second, we harvested whole eyes, 24 and 48 hrs after infection, and obtained viable bacterial counts by plating serial dilutions of whole eye homogenates. The results showed significantly higher bacterial counts from the *Lum* ^−/−^ whole eye homogenates (Fig. 2B).

### Inflammatory Cell Infiltration in Infected Corneas

**Figure 3:**
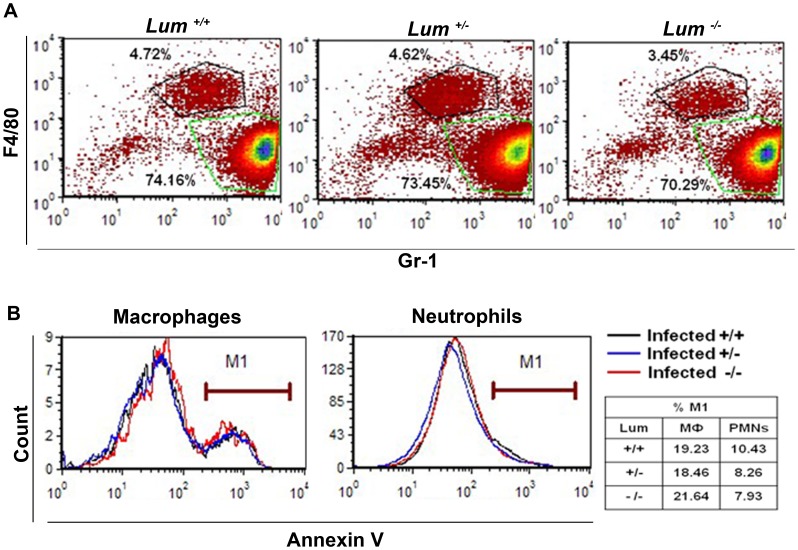
Flow cytometry of 24 hrs infected corneas shows reduced inflammatory cell percentages in *Lum* ^−/−^ corneas. Total cells from control and infected corneas per genotype (n = 5) were subjected to FACS analysis. Cells were immunostained with F4/80 (macrophage or MΦ marker), Gr-1 (neutrophil or PMN marker) (A) and with APC-Annexin V for detecting apoptotic cells (B). The percentage of MΦs and PMNs with respect to total cells was reduced in the *Lum* ^−/−^ pool to 3.45% and 70.29% respectively, compared to the *Lum ^+/+^* and *Lum^+/−^* controls. There was a decrease in the percentage of AnnexinV^+^cells in the *Lum* ^−/−^ PMN population.

Increased survival of the bacterium in the *Lum* ^−/−^ cornea could be due to impaired functions of macrophages and neutrophils and/or poor recruitment of inflammatory cells. We used flow cytometry to obtain a total count of PMNs and macrophages from 5 animals after 24 hrs infection. Total cells from 5 pooled corneas per genotype were immunostained with F4/80 (macrophage marker), Gr-1 (PMN marker) and binding of AnnexinV as an indicator of apoptotic cells. Five uninfected corneas per genotype used as controls showed minimal infiltrates and no genotype-dependent differences (data not shown). After infection, the total cell count was similar in heterozygous (164×10^4^/ml) and wild types (186×10^4^/ml), but lower in the knockout (84×10^4^/ml) pool. We quantified the F4/80^high^ Grl^low^ (macrophages) and the F4/80 ^low^ Gr1^high^ (PMNs) cells in each pool and expressed these as a percentage of total cells. The percentages of PMNs and macrophages were comparable in the wild type and heterozygous pool, but markedly reduced in the infected *Lum* ^−/−^ pool (Fig. 3A). To determine if leukocyte apoptosis was affected by lumican-deficiency, we quantified AnnexinV^+^ cells in the total cell population and cells gated for F4/80 and GR-1 (Fig. 3B). There was a slight reduction in the percentage of AnnexinV^+^ cells in the PMN population, and no significant difference in the macrophage population in *Lum* ^−/−^ compared to the heterozygous and wild type pool.

We showed previously that CD14-dependent phagocytosis and bacterial clearance by macrophages *in vitro* was reduced in *Lum* ^−/−^ mice [5]. To determine if *Lum* ^−/−^ PMNs were also defective in bacterial killing, we incubated peritoneal PMNs and bacteria together and measured viable CFU recovered from lysed cells and the supernatant. We used the *P. aeruginosa* strain 808 to assay *in* *vitro* killing, as this strain is known to be taken up by the host in a CD14 dependent manner and our study with *Lum* ^−/−^ macrophages showed its dependence on lumican [5]. The yield of viable CFU was similar from *Lum* ^+/−^ and *Lum* ^−/−^ PMNs at each time point (Fig. S1). Thus, *in* *vitro* killing by peritoneal neutrophils was not adversely affected by lumican deficiency. Taken together, increased bacterial yield in the infected *Lum* ^−/−^ corneas could be due to a combination of reduced macrophage and PMN infiltration and phagocytosis deficiency in macrophages, even though bacterial killing by PMNs was not affected.

### Lumican Expression in *P. aeruginosa* Infected Corneas

**Figure 4:**
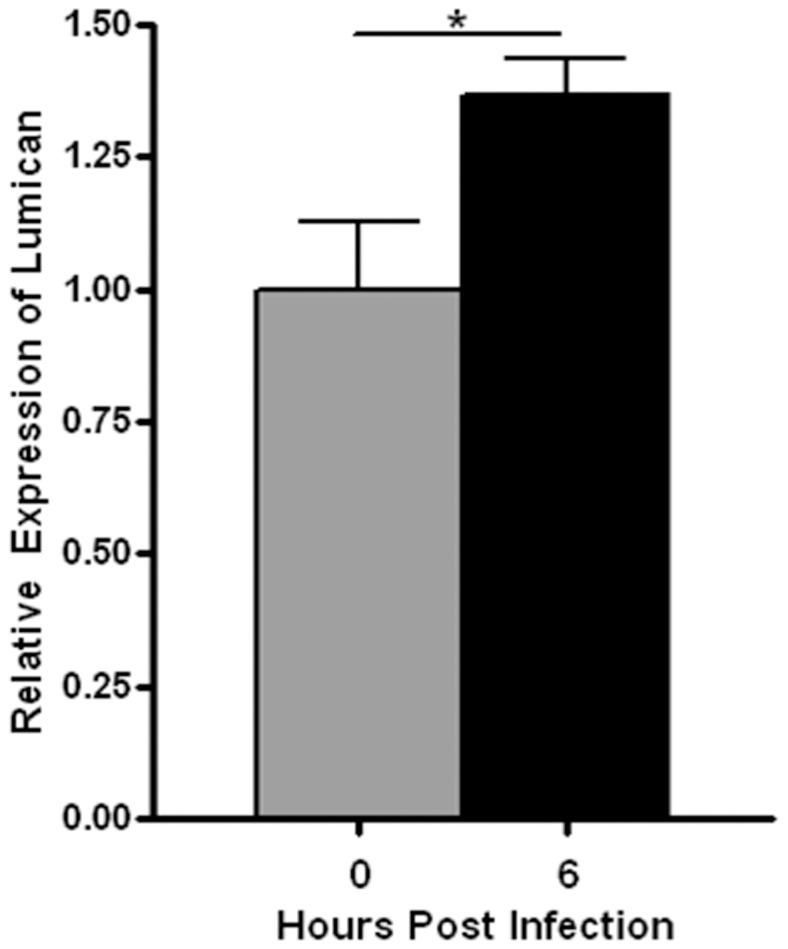
Increased lumican expression in *Lum ^+/−^* corneas 6 hr post infection. Lumican transcript relative to18S RNA was significantly increased in *Lum* ^+/−^ corneas 6 hrs after infection as determined by qRT-PCR (n = 4). * *p*≤0.05.

We have previously shown that lumican expression is up regulated during inflammation in the colon and *in vitro* in fibroblasts after treatment with LPS or IL-1β [3], [33]. We questioned whether expression of lumican is similarly up regulated during *P. aeruginosa* infections of the cornea. By qRT-PCR we detected increased lumican transcript in *Lum* ^+/−^ corneas by 6 hrs after infection, indicating up regulation of lumican expression in resident corneal cells before inflammatory cell infiltrations, underscoring a role for lumican during the development of host response to pathogenic bacteria (Fig. 4).

### Induction of Inflammation Mediators after Infection

**Figure 5:**
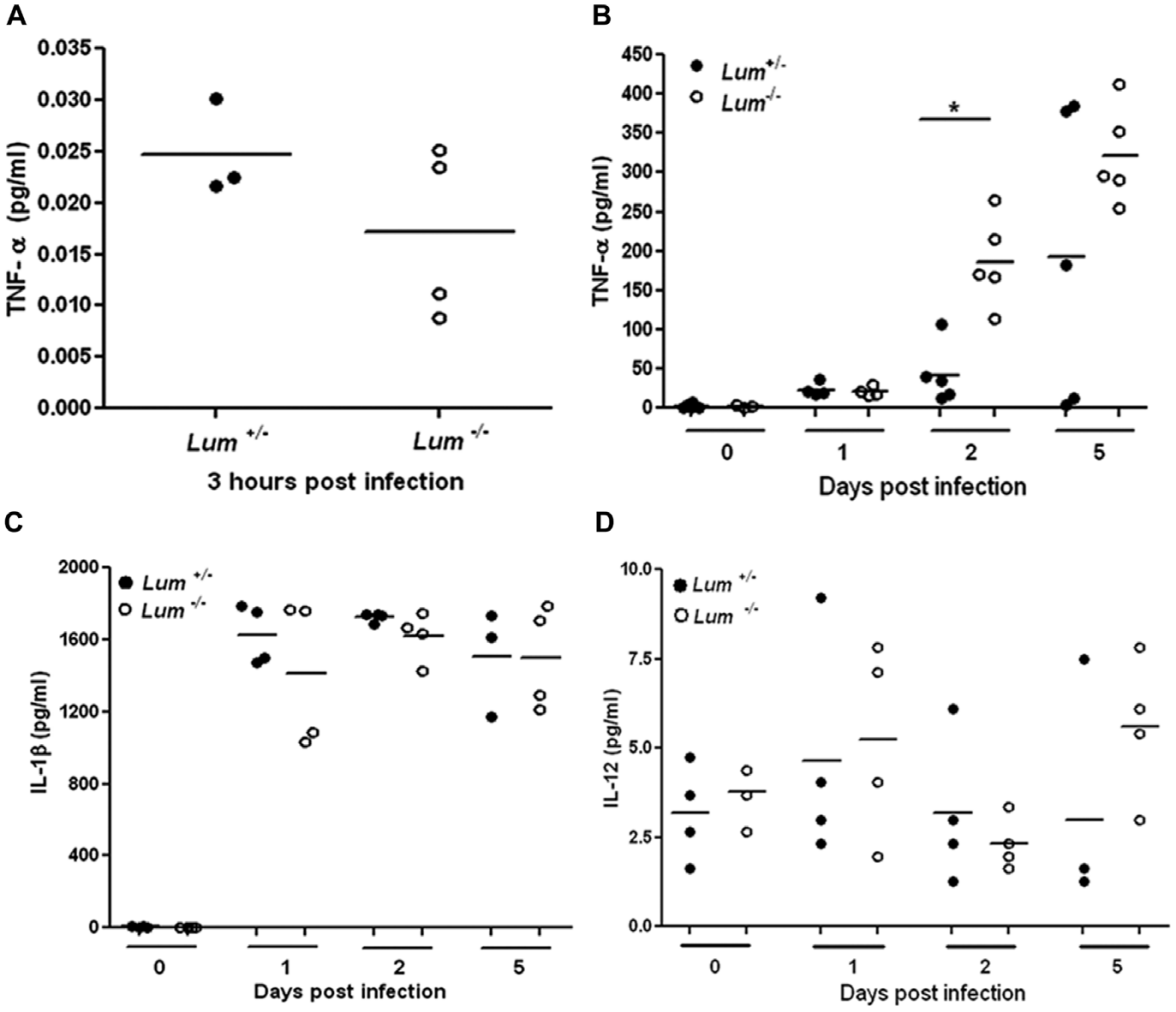
Proinflammatory cytokines measured by ELISA in *P.aeruginosa* infected corneas. TNF-α level was low and comparable in *Lum* ^+/−^ and *Lum* ^−/−^ corneal protein extracts 3 hrs after infection (A) but significantly higher in *Lum* ^−/−^ infected corneas 2 days after infection (B). IL-1β was induced comparably in *Lum* ^+/−^ and *Lum* ^−/−^ infected corneas for up to 5 days after infection (C) and IL-12 (D) was detected at very low levels in infected corneas of both genotypes. * *p*≤0.05.

To determine if induction of pro-inflammatory cytokines and chemokines in response to *P. aeruginosa* infection was affected adversely by lumican-deficiency, we measured selected cytokines and chemokines in total protein extracts of control and infected corneas. Three hours after infection, when neutrophils have yet to infiltrate the cornea, TNF-α concentration was close to baseline in infected corneas of both genotypes (Fig. 5A). By 24 hrs after infection the level of TNF-α had increased to similar extents in *Lum* ^+/−^ and *Lum* ^−/−^ corneas (94 and 84 pg/ml on average, respectively). However, by 2 days after infection the TNF-α level in *Lum* ^−/−^ infected corneas was significantly higher - 930 pg/ml versus 210 pg/ml, and by 5 days it was 1607 pg/ml in infected *Lum* ^−/−^ versus 963 pg/ml in wild type corneas (Fig. 5B).

IL-1β, a pro-inflammatory cytokine activated by NLRP inflammosome signals, has been shown to be elevated in *P. aeruginosa* keratitis [34], [35]. Mature IL-1β was undetectable before corneal infection, and induced comparably to 1200–1600 pg/ml in the *Lum* ^+/−^ and the *Lum* ^−/−^ infected corneas for up to 5 days after infection (Fig. 5C). IL-12 is produced by macrophages and NK cells in some bacterial and viral infections and facilitates the development of T_H_1 differentiation [36]. We tested the induction of IL-12 after infection. Using the IL-12 p70 ELISA, which recognizes the IL-12 p35/p40 heterodimer specifically, we detected very low levels of the IL-12 heterodimer in infected corneas of all genotypes (Fig. 5D), while measuring the p40 subunit, common to IL-12 and IL-23, another study reported a small increase in *P. aeruginosa* infected B6 mouse corneas [37].

**Figure 6:**
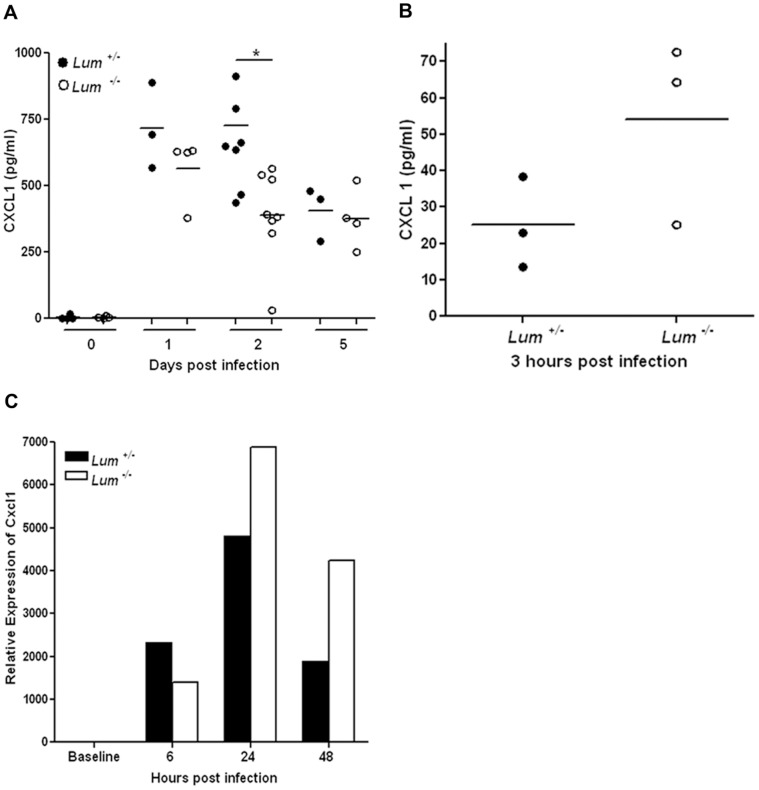
Differential regulation of CXCL1 in infected *Lum ^+/−^* and *Lum ^−/−^* corneas. CXCL1 measured by ELISA increased 24 hrs after infection in both genotypes, with its level being consistently lower in the *Lum* ^−/−^ corneal extracts - the difference being statistically significant at the 2 d.p.i time point (A). Three hours after infection CXCL1 levels were comparably low in both genotypes (n = 3) (B). The *Cxcl1* transcript measured by qRT-PCR in quadruplicates/animal was increased over baseline 6 hrs post infection; by 24–48 hrs post infection *Lum* ^−/−^ corneas showed higher levels of *Cxcl*1 compared to *Lum* ^+/−^ (C). * *p*≤0.05.

The CXC chemokine ligand 1 (CXCL1/GRO-α), produced by fibroblasts and macrophages, is a key chemoattractant for neutrophils in inflamed corneas [38], [39]. CXCL1 was elevated within 24 hrs of infection in both genotypes; however, its level was consistently lower in infected *Lum* ^−/−^ corneas compared to *Lum* ^+/−^ corneas. Average levels in *Lum^+/−^* and *Lum^−/−^* infected corneas were 717 and 566 pg/ml after one day, 705 and 500 pg/ml after 2 days, and 408 and 377 pg/ml after 5 days of infection, respectively (Fig. 6A). Three hours after infection, CXCL1 was slightly increased in both genotypes without significant differences (Fig. 6B). To determine if the decrease in CXCL1 at the later stages of infection was due to transcriptional differences in regulation in the absence of lumican, we determined *Cxcl1* expression by real time qRT-PCR in total RNA extracted from *Lum* ^+/−^ and *Lum* ^−/−^ infected corneas. Six hrs after infection *Cxcl1* expression was higher in the *Lum* ^+/−^ compared to *Lum* ^−/−^ corneas. However, 24 and 48 hrs after infection *Cxcl1* transcript levels were significantly higher in the knockout infected corneas - a trend that was just the opposite of final chemokine levels measured by ELISA (Fig. 6C). This dichotomy between protein and transcript levels, suggesting translational regulation of *Cxcl1* as a function of lumican is discussed later.

### Inflammatory Cell Infiltration and Tissue Damage in Infected *Lum* ^−/−^ Corneas

**Figure 7:**
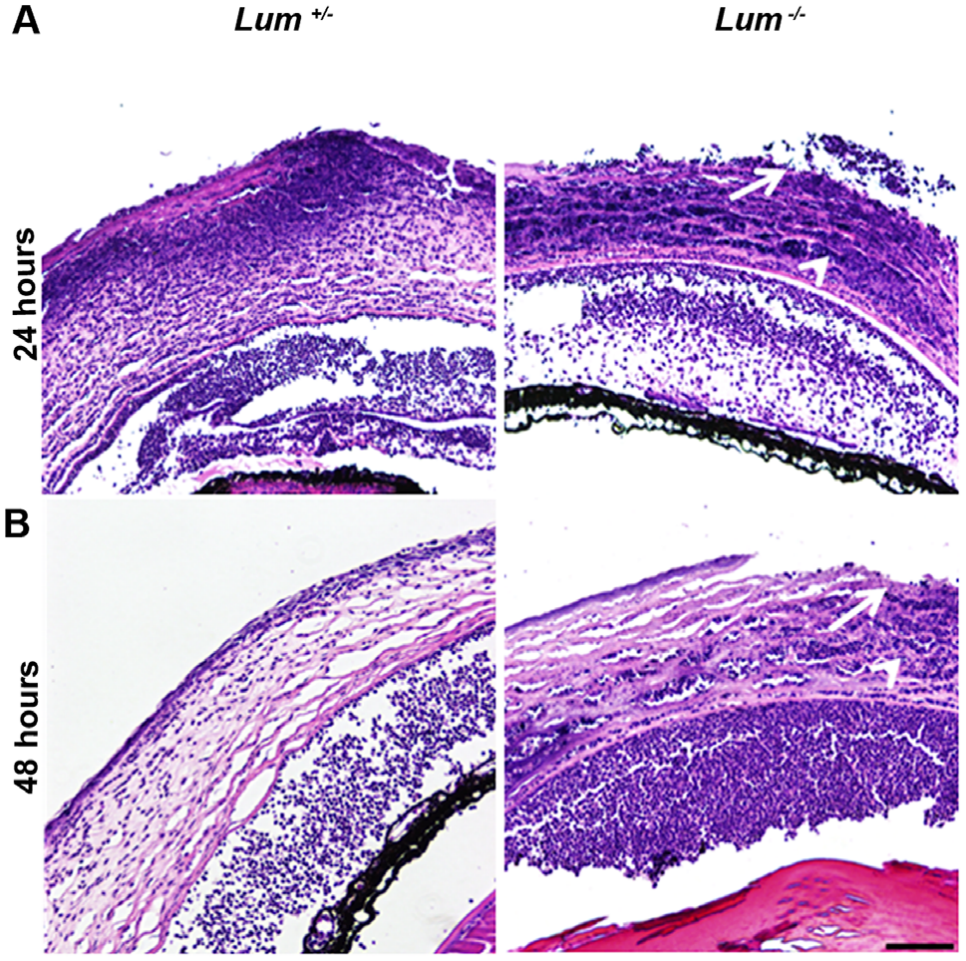
Increased tissue damage in infected corneas of *Lum* ^+/−^ and *Lum ^−/−^* mice. Paraffin-embedded sections of eyes 24 hrs (A) and 48 hrs (B) after infection were stained with H and E. To examine tissue damage in mice with comparable disease all infected animals used for histology had an initial disease score of 2 to 3 and showed PMN infiltrations in the cornea and anterior chamber. The *Lum ^−/−^* infected corneas showed large areas of epithelial ulcerations (arrow) and stromal damage (arrowhead). Scale bar, 100 µm.

To specifically examine tissue damage in infected *Lum* ^+/−^ and *Lum* ^−/−^ mice by histology, we harvested mice 24 and 48 hrs after infection with disease scores of 2 and 3, respectively. Tissue damage at the 24 hrs time point was similar in *Lum* ^+/−^ and *Lum* ^−/−^ corneas (Fig. 7A). By 48 hrs the *Lum* ^−/−^ corneas showed large areas of epithelial ulcerations, and increased damage of the stroma (Fig. 7B). Also, there was very little damage of the corneal endothelium and no obvious edema in infected corneas of either genotype, further underscoring lumican involvement in bacterial infections of the stroma directly.

## Discussion

Here we showed that in a *P*. *aeruginosa* keratitis model, the *Lum* ^−/−^ mice responded differently from control mice in a number of ways such that their recovery from keratitis was compromised. The *Lum* ^−/−^ mice showed poor resolution of keratitis, increased bacterial survival, and differences in pro-inflammatory cytokines, chemokine ligands and inflammatory cell infiltrates. Our previous studies show that lumican is a structural extracellular matrix protein that also interacts with macrophage and neutrophil cell surfaces and regulates TLR4 mediated LPS sensing specifically [3]–[5], [33]. Several aspects of keratitis, such as poor bacterial clearance, induction of pro-inflammatory cytokines and poor disease resolution manifested by *Lum* ^−/−^ mice are features shared by, and more pronounced in mice lacking major LPS signaling intermediates, TLR4 or MyD88 or transgenic Mafia mice after macrophage depletion [13], [28]. This is consistent with our findings that lumican is a modulator of the LPS response.

TNF-α is a pro-inflammatory cytokine produced by a variety of antigen presenting cells of innate immunity, and by CD4+ T_H_1 cells of adaptive immunity [40]. As TNF-α also leads to further activation of NF-κB and amplification of inflammatory responses, it can contribute to harmful corneal damages if uncontrolled. Three hrs after infection TNF-α was close to baseline in both genotypes, indicating that early innate immune response from resident corneal cells was not affected by lumican-deficiency. However, TNF-α was significantly higher in the *Lum* ^−/−^ corneas by 2 days after infection. In our earlier study, we reported that in a LPS-stimulated septic shock model the *Lum* ^−/−^ mice produced lower levels of TNF-α, and isolated macrophages stimulated with LPS *in* *vitro* also produced lower levels of TNF-α due to poor LPS sensing. The answer to why we see an increase in TNF-α in *Lum* ^−/−^ corneas at the later stages of infection with live bacteria may not be a simple one. One explanation for this difference in the *Lum* ^−/−^ mouse response to LPS versus live *P. aeruginosa* is the fact that host response to live bacteria involves multiple PAMPs and not LPS alone. The observation that this increase in TNF-α occurs later in disease by 48 hrs indicates that once the initial delay in TLR4 signals [3], [5], and β2 integrin mediated neutrophil migration [4], have been overcome in the lumican-deficient mice, poor bacterial clearance contributes to continued induction of innate immune responses via other TLRs, such as TLR5 mediated response to flagellin [41], resulting in elevated pro-inflammatory cytokines.

The CXC chemokine ligand 1, CXCL1 or keratinocyte-derived chemokine, KC, produced by epithelial cells, fibroblasts, PMNs and macrophages, is a major chemoattractant for PMNs. CXCL1 was shown to be temporally up regulated in an LPS treated sterile keratitis model in the mouse [38], but its increase in lumican-deficient corneas was lower than that of wild types. It has been shown by others that in the sterile LPS-keratitis model, CXCL1 is up regulated within 3 hrs of injury, with maximum induction 24 hrs after injury and subsiding by 72 hrs [38]. In agreement with these sterile keratitis findings, after infection with *P. aeruginosa*, we also noted a rapid increase in CXCL1. Furthermore, by 48 hrs of infection the increase in CXCL1 levels in *Lum* ^−/−^ infected corneas was much lower than *Lum* ^+/−^. However, *Cxcl1* expression was higher in the *Lum* ^−/−^ corneas at the later stages of infection, suggesting that reduced CXCL1 levels in the *Lum* ^−/−^ infected corneas may contribute to a feedback up regulation of gene expression. Similarly, CXCL1 levels in the sterile LPS-keratitis model was reportedly increased in the anterior chamber of mice deficient in lumican and keratocan, another corneal proteoglycan. It was proposed that in the absence of these proteoglycans, the chemokine is not retained in the cornea and accumulates in the anterior chamber [14]. Thus, our observation that the *Lum* ^−/−^ infected corneas harbored lower levels of the chemokine compared to *Lum* ^+/−^ corneas could be due to poor retention, or increased degradation of the chemokine.

Another important pro-inflammatory cytokine induced during infections is IL-1β; its expression is induced by NF-κB transcription factors. At the protein level, pro-IL-1 β is cleaved to release the mature IL-1β by caspase-1 activated by NLRP inflammosome signals in the presence of bacterial toxins [42]. Mature IL-1β had increased dramatically in the cornea by 24 h of infection, and this was not significantly affected by lumican deficiency. Thus, it appears that lumican may not regulate inflammosome mediated elevation of mature IL-1β. Overall, *Lum* ^+/−^ and *Lum* ^−/−^ infected corneas showed comparable increases in IL-1β. While TNFα and CXCL1 had increased in both genotypes by 48 hrs, coinciding with the timing of inflammatory cell influx, the increase in these two cytokines were significantly lower in *Lum* ^−/−^ infected corneas compared to their wild type allele carrying counter parts.

The increased severity of keratitis seen in the *Lum* ^−/−^ mice with *P*. *aeruginosa* ATCC19660 may be most closely linked to poor bacterial clearance by phagocytosis in the *Lum* ^−/−^ mice. Thus, failure to clear the bacteria in the *Lum* ^−/−^ cornea is likely to contribute to increased bacterial toxins, increased host innate immune response and production of TNF-α and tissue damaging proteases by the host. Interestingly, we also found poor bacterial clearance in the *Lum* ^−/−^ mice in an earlier lung infection model; however lung tissue TNF-α level was comparable to wild types [5]. This may be partly due to intrinsic differences between the cornea as a naturally macrophage poor tissue, and the lungs rich in resident alveolar macrophages. *In vitro*, we have shown previously that the *P. aeruginosa* ATCC19660 and the 808 strain were not taken up and killed by *Lum* ^−/−^ peritoneal macrophages as effectively as wild types [5]. By contrast, here when we examined *in* *vitro* killing by PMNs, we found no major difference between *Lum* ^−/−^ and *Lum* ^+/−^ mice. So why is there an increased bacterial load in *Lum* ^−/−^ keratitis? Several factors may be at play here. First, by flow cytometry we found that 24 hrs after infection, macrophage and PMN percentages were lower in *Lum* ^−/−^ corneas. Second, while *Lum* ^−/−^ PMNs are competent in bacterial killing, *Lum* ^−/−^ macrophages are phagocytosis-impaired. This must impact bacterial clearance, as well as antigen presentation, recruitment/proliferation of other lymphocytes and corneal immune privilege. Future temporal analyses of these cell populations in the infected cornea will address these questions.

In conclusion, lumican deficiency leads to poor clearance of *P*. *aeruginosa* infections of the cornea, continued production of pro-inflammatory cytokines and inefficient resolution of inflammation.

## Supporting Information

Figure S1 ***In vitro*** **killing by peritoneal PMNs.** *In vitro* killing was determined by measuring viable CFU in the supernatant and cell lysate of *Lum ^+/−^* and *Lum ^−/−^* PMNs. There was no significant difference in the killing capabilities of PMNs from both genotypes.
(TIF)
