# Supplementary figures and images for: Extracellular Matrix Protein Lumican Promotes Clearance and Resolution of Pseudomonas aeruginosa Keratitis in a Mouse Model
Source: PLoS One. 2013 Jan 24;8(1):e54765. doi: 10.1371/journal.pone.0054765 (PMC3554612; doi:10.1371/journal.pone.0054765)

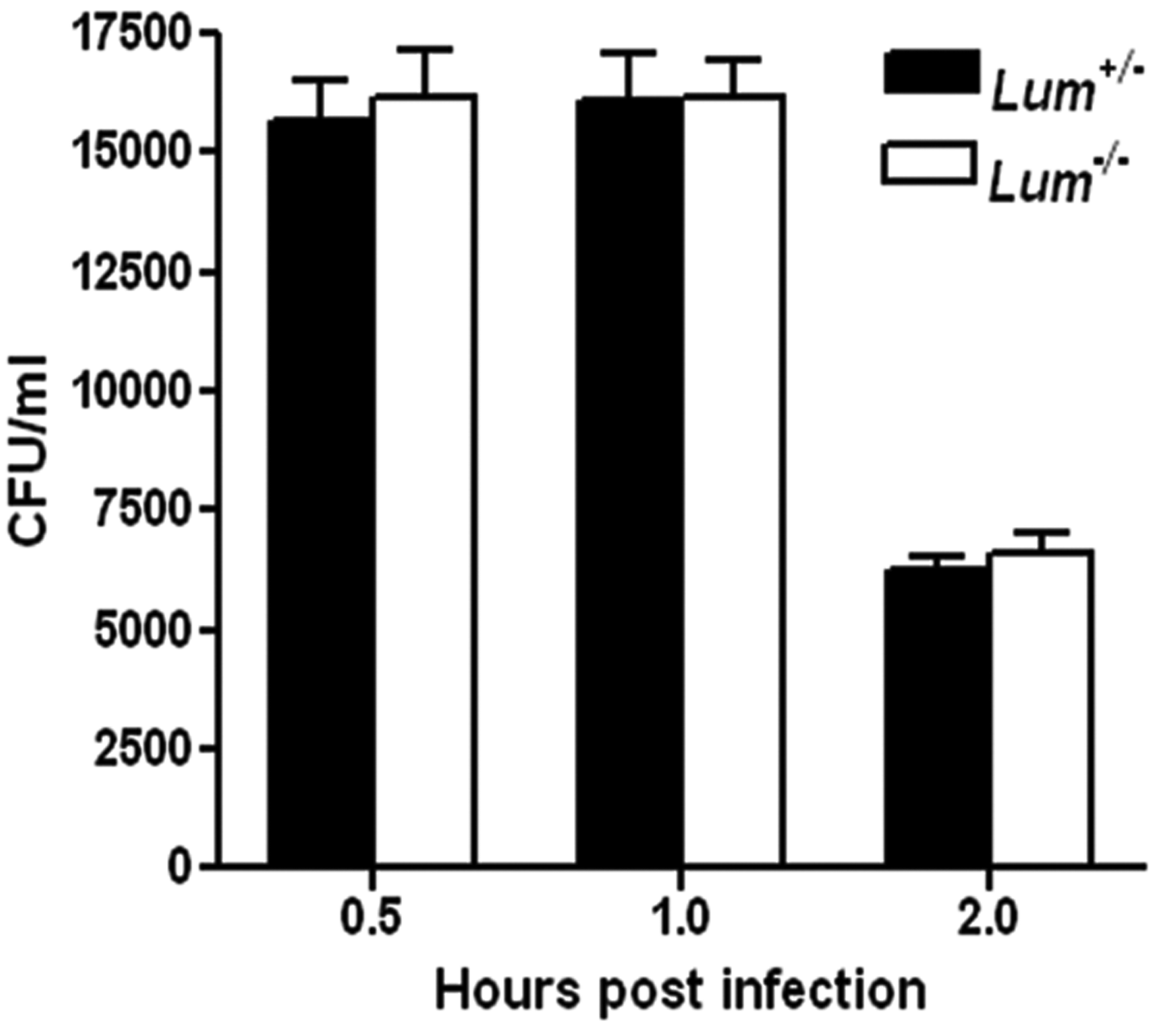

Supplement: Figure S1 — In vitro killing by peritoneal PMNs. In vitro killing was determined by measuring viable CFU in the supernatant and cell lysate of Lum +/− and Lum −/− PMNs. There was no significant difference in the killing capabilities of PMNs from both genotypes. (TIF) [file pone.0054765.s001.tif]
